# How AMPK and PKA Interplay to Regulate Mitochondrial Function and Survival in Models of Ischemia and Diabetes

**DOI:** 10.1155/2017/4353510

**Published:** 2017-12-17

**Authors:** Jingdian Zhang, Yumeng Wang, Xiaofeng Liu, Ruben K. Dagda, Ying Zhang

**Affiliations:** ^1^Department of Neurology and Neuroscience Center, First Hospital of Jilin University, Xinmin Street No. 71, Changchun 130000, China; ^2^Department of Physiology, College of Basic Medical Sciences, Norman Bethune Health Science Center, Jilin University, Xinmin Street No. 126, Changchun 130000, China; ^3^Neuroscience Research Center, The First Hospital of Jilin University, East Democracy Street No. 519, Changchun 130000, China; ^4^Department of Pharmacology, University of Nevada, Reno School of Medicine, Mailstop 318, Manville Health Sciences Building 19A(Office)/18, Reno, NV 89557, USA

## Abstract

Adenosine monophosphate-activated protein kinase (AMPK) is a conserved, redox-activated master regulator of cell metabolism. In the presence of oxidative stress, AMPK promotes cytoprotection by enhancing the conservation of energy by suppressing protein translation and by stimulating autophagy. AMPK interplays with protein kinase A (PKA) to regulate oxidative stress, mitochondrial function, and cell survival. AMPK and dual-specificity A-kinase anchoring protein 1 (D-AKAP1), a mitochondrial-directed scaffold of PKA, interact to regulate mitochondrial function and oxidative stress in cardiac and endothelial cells. Ischemia and diabetes, a chronic disease that increases the onset of cardiovascular diseases, suppress the cardioprotective effects of AMPK and PKA. Here, we review the molecular mechanisms by which AMPK and D-AKAP1/PKA interplay to regulate mitochondrial function, oxidative stress, and signaling pathways that prime endothelial cells, cardiac cells, and neurons for cytoprotection against oxidative stress. We discuss recent literature showing how temporal dynamics and localization of activated AMPK and PKA holoenzymes play a crucial role in governing cellular bioenergetics and cell survival in models of ischemia, cardiovascular diseases, and diabetes. Finally, we propose therapeutic strategies that tout localized PKA and AMPK signaling to reverse mitochondrial dysfunction, oxidative stress, and death of neurons and cardiac and endothelial cells during ischemia and diabetes.

## 1. Introduction

AMPK is a heterotrimeric holoenzyme that consists of a catalytic subunit (*α*) bound to two regulatory subunits (*β* and *γ*). Due to the diverse arrangement of different isoforms of the three subunits of the AMPK holoenzyme, there can exist up to 12 different AMPK holoenzymes in different tissues, which add a layer of complexity AMPK-mediated regulation of critical physiological functions in eukaryotes [1, 2].

AMPK activity is induced by a high ratio of intracellular AMP to ATP levels. AMP directly binds to the regulatory subunit of AMPK to facilitate AMPK phosphorylation via the upstream protein kinase LKB1 [3]. Upon phosphorylation by LKB1, activated AMPK restores intracellular energy levels by inhibiting ATP-consuming biosynthetic pathways and by stimulating catabolic ATP-regenerating processes. Furthermore, AMPK is a redox-sensing kinase that regulates cellular bioenergetics. Indeed, mitochondrial-derived free radicals can activate AMPK through LKB1 without altering the ratio of AMP/ATP [4, 5].

**Figure 1:**
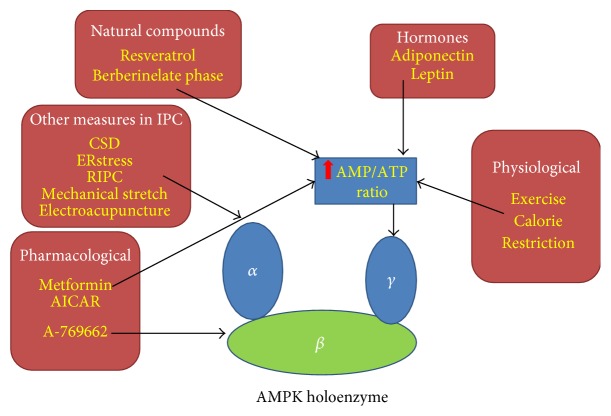
Non-redox-dependent physiological, pharmacological, natural compounds and other conditions that activate AMPK in ischemic preconditioning. This figure highlights some of the well-established and newly discovered AMPK activators or conditions that have benefits in ischemic preconditioning. Pharmacological activators such as metformin and AICAR, some natural compounds, and physiological situations such as exercise and calorie restriction can activate AMPK by increasing the AMP : ATP ratio (shown in the blue rectangle), causing AMP to bind to the *γ*-subunit. However, a subset of conditions or pharmacological compounds can stimulate AMPK activation in IPC via other mechanisms, such as activated upstream kinases of the *α*-subunit (shown in the rounded rectangle named “other measures in IPC”), or binding directly to the *β*-subunit (A769662).

AMPK can be activated via two different mechanisms: (1) phosphorylation of threonine 172 (Thr172) by upstream kinases or via autocatalytic-mediated phosphorylation or (2) by binding of AMP to AMPK. Specifically, LKB1, CaMKKb, or TAK1 can phosphorylate Thr172 within the activation loop of the *α*-subunit when AMP binds to the *γ*-subunit [2, 6–9]. All the aforementioned upstream kinases, as well as levels of AMP induced by acute/chronic stress, can activate AMPK to activate downstream biological functions [10].  Figure 1 and Supplementary Table S1 show ways that AMPK can be activated pharmacologically in a ROS-dependent manner.

The phosphorylation and subsequent activation of AMPK elicit the following physiological functions depending on the type and intensity of toxic insult and oxidative stress: (1) regulation of metabolism and glucose uptake, (2) modulation of protein synthesis and cellular growth by inhibiting the mammalian target of rapamycin (mTOR) pathway, and (3) initiating autophagy during starvation or by specific conditions that induce severe stress [10]. In the context of metabolic stress (metabolic crisis), most cells or organs benefit from activation of AMPK signaling with the exception of brain tissue which has shown modest cytoprotection or detrimental outcomes during ischemia [11]. Activation of AMPK signaling regulates cell metabolism. For instance, AMPK stimulates fatty acid oxidation [12], mitochondrial biogenesis [13–15], glucose transporter type 4 (GLUT4) translocation, and glucose uptake [16–18], while inhibiting protein synthesis, gluconeogenesis, and fatty acid and cholesterol synthesis, [19–21] to increase ATP levels in order to reduce oxidative stress caused by metabolic crisis [22].

Mitochondria not only are regulators of oxidative phosphorylation and calcium homeostasis but also regulate a variety of converging cell death signaling pathways that activate programmed cell death. The mechanisms by which AMPK regulates mitochondrial function and cell survival have recently gained interest, but this area of research is still in its infancy. Recent evidence shows that AMPK has the capacity to regulate cellular bioenergetics, cell death, and mitochondrial structure/function and dynamics by interacting with PKA or, in parallel, during chronic or acute oxidative stress. In this review, we present evidence on the molecular mechanisms by which AMPK and PKA coregulate mitochondrial function and structure in cardiac cells and neurons, two cell types targeted in ischemia and in diabetes, a chronic disease that increases the risk for developing cardiovascular diseases (CVDs) and stroke. Indeed, the current global incidence of obesity and type 2 diabetes has increased in part due to a combination of sedentary lifestyle and high-calorie diets. Secondly, we highlight the importance of how the temporal dynamics and intracellular localization of activated AMPK and PKA holoenzymes play a critical role in regulating oxidative stress and cellular bioenergetics in cardiac cells, endothelial cells, and neurons.

## 2. AMPK Regulates Mitochondrial Dynamics, Function, and Structure

Since the mitochondria act as major “power plants” of eukaryotes, exploring the interaction between AMPK and mitochondria will shed more insight as to how cells maintain overall energy homeostasis and mitochondrial quality control [23, 24]. In addition, AMPK promotes mitochondrial biogenesis (generation of new mitochondria) in various tissues. For instance, by stimulating PGC1-*α* and NRF1/2 expression, AMPK participates in parallel to the E3 ubiquitin ligase Parkin to govern mitochondrial biogenesis, presumably as a compensatory response to preserve mitochondrial homeostasis [3, 25–27].

Mitochondrial fission/fusion and movement (trafficking) is regulated by protein phosphorylation imparted by a variety of ser/thr kinases including PKA and AMPK [28, 29]. Recently, there have been an increasing number of studies that show that AMPK regulates mitochondrial structure and function [5, 26, 30–32]. For instance, in neurons, AMPK signaling regulates anterograde transport of mitochondria in the axons of depolarized neurons [33]. In nonneuronal cells, AMPK signaling supports mitochondrial fragmentation (fission) of oxidatively damaged mitochondria by activating the fission modulator dynamin-related protein 1 (Drp1). In support of this concept, a landmark study recently showed that AMPK facilitates mitochondrial fission induced by acute treatment with the complex I inhibitor rotenone. Conversely, U2OS cells deficient for both the *α* and *β* subunits of AMPK are recalcitrant to mitochondrial fragmentation induced by mitochondrial-damaging compounds rotenone and antimicyn-A [29]. Mechanistically, AMPK activation promotes mitochondrial fission by phosphorylating mitochondrial fission factor (MFF) and by activating ULK [29, 34].

Cells require a minimum level of high-quality mitochondria to produce the necessary energy to thrive. Oxidatively damaged and effete mitochondria are continuously targeted for autophagolysosomal-mediated degradation via a selective physiological process termed mitophagy [35, 36]. Importantly, an increase in the turnover of mitochondria via mitophagy allows for not only the removal of damaged/effete mitochondria but also for the induction and integration of mitochondrial biogenesis pathways to restore mitochondrial levels [23]. Given that mitochondrial fission facilitates the induction of mitophagy [37], stimulating Drp1-dependent mitochondrial fission via AMPK signaling facilitates the removal of mitochondria via mitophagy [29, 34] in order to maintain a level of healthy mitochondria.

### 2.1. AMPK Regulates Mitochondrial Quality Control

AMPK is a *bona fide* regulator of mitochondrial dynamics, mitochondrial autophagy, and biogenesis. Indeed, when metabolic stress persists, damaged mitochondria will lead to robust Drp1-dependent mitochondrial fragmentation which can facilitate apoptosis [38, 39]. As mentioned before, activated AMPK triggers mitochondrial fission, at least in part via phosphorylation of MFF, which consequently activates the kinase ULK1 to initiate autophagy. Thus, AMPK may couple mitochondria fission to mitophagy in a continuous feed-forward cycle to maintain the levels of high-quality mitochondria and energy in the cell. A study by Toyama et al., 2016 shed insight on the mechanism by which AMPK activates fission in human U2OS osteosarcoma cells, SV40-immortalized murine embryonic fibroblasts (MEFs), and layer 2/3 cortical pyramidal neurons [29]. On the contrary, other research reports suggest that metformin can inhibit Drp1-mediated mitochondrial fission in endothelial cells of streptozocin- (STZ-) induced diabetic ApoE^−/−^ mice and in adipose tissue of STZ-induced diabetic WT mice [40, 41]. Overall, these studies suggest that AMPK can have opposing effects on mitochondrial fission/fusion, a phenomenon that likely depends on the bioenergetic status and levels of oxidative stress in the cell.

### 2.2. MFF Level Regulates AMPK-Mediated Fission

One possibility is that the levels of MFF may govern the ability of AMPK to regulate mitochondrial dynamics. MFF was observed to be in low abundance in human umbilical vein endothelial cells (HUVECs) and human vascular smooth muscle cells (HVSMCs) [41]. In addition, AMPK has been found to directly mediate mitochondrial fission via MFF in response to energy stress [29]. Neurons contain intermediate levels of MFF level while MFF is undetectable in astrocytes and brain endothelial cells or other heart or muscle tissues according to the Human Protein Atlas Program. In peripheral tissues, MFF levels are low including myocytes, hepatocytes, endothelial cell, astrocytes, and in renal glomeruli. AMPK regulates mitochondrial fission through MFF in U2OS cells [29]. Interestingly, AMPK may induce mitochondrial fusion in cells that are chronically stressed or in certain pathological conditions. For instance, endothelial cells from streptozotocin- (STZ-) induced diabetic ApoE^−/−^ mice treated with metformin show an inhibition of Drp1-mediated mitochondrial fission [41]. In addition, AMPK activation inhibits high glucose-induced Drp1-mediated mitochondrial fission in epididymal adipose tissue both *in vitro* and in *in vivo* [40]. Supplementary Table S2 gives a comprehensive list of pharmacological compounds that confer protection in cells by activating AMPK in a non-ROS manner (Supplementary Materials online, Table S2). Adipose tissue, another tissue with low levels of MFF, benefits from the inhibition of mitochondrial fission via AMPK activation in hyperglycemic conditions. Overall, it is likely that the levels of oxidative stress and the extent of mitochondrial damage dictate whether AMPK promotes fusion or fission. Future studies that elucidate the mechanisms by which oxidative stress regulates AMPK-mediated mitochondrial fission/fusion are warranted.

During physiological conditions, neurons require a continuous distribution of mitochondria across long distances including dendrites, axons, and synapses in order to meet end-to-end energy requirements, neurotransmission, dendrite development, and efficient Ca^+2^buffering [42]. Given that AMPK regulates mitochondrial movement in neurons [33], it is conceivable that neurons contain a higher level of MFF to achieve a minimum level of mitochondrial trafficking required in very extensive and vast neuronal networks. Hence, AMPK activation induces diverse effects on mitochondria dynamics which partly depends on levels of endogenous MFF across different tissues.

## 3. Role of AMPK as a Redox Sensor of Mitochondrial ROS

The mitochondria are the main generators of cellular ATP production via oxidative phosphorylation. However, if protein complexes embedded in the inner mitochondrial membrane or enzymes which catalyze cascade of redox reactions in ETC lose their tight association with the IMM or are damaged, electrons will leak and consequently generate detrimental levels of ROS by interacting with oxygen [43]. For instance, electrons can leak from complex I and react with oxygen to generate superoxide [44, 45]. Indeed, the mitochondria are the primary source of intracellular ROS levels and contribute up to 95% of total ROS levels [46].

Transient and moderate levels of ROS during preconditioning will induce cytoprotective responses by regulating either protein function and/or gene expression [47]. Recent ischemia preconditioning studies show that ROS-mediated activation of AMPK is associated with resistance against ischemia. A myriad of different toxic and physiological stimuli that activate AMPK can exert cytoprotection in models of ischemia. For instance, Supplementary Table S1 shows that hydrogen peroxide, hypoxic conditions, low glucose concentrations, thyroid hormone, and many drugs can activate AMPK in a ROS-dependent manner despite a stable ratio of AMP/ATP [4, 5, 30, 31, 48, 49] (Supplementary Materials online, Table S1).

### 3.1. Redox-Activation of AMPK Regulates Cell Survival during Ischemia

Sublethal hypoxic and ischemic events (ischemic preconditioning) or some drugs can enhance the tolerance of tissues and organs, to subsequent lethal injury induced by hypoxia, ischemia, and ischemia-reperfusion [50]. The induction of this ischemic tolerance can be achieved by three major approaches: (1) ischemic conditioning, (2) hypoxic conditioning, and (3) chemical conditioning [51]. Interestingly, hypothermic preconditioning exerts a more pronounced cardioprotective effect than ischemic preconditioning [52]. AMPK can be activated by ischemic preconditioning as well [4, 5, 30, 48, 49, 52–54]. In this context, mitochondrial-derived ROS induced by different preconditioning paradigms leads to activation of AMPK and induces resistance to subsequent lethal injury. Mechanistically, ROS scavengers or compound C can both diminish this protection alone suggesting that ROS is a modulator of AMPK-dependent cytoprotection against ischemia [30, 48, 53]. The fact that cells deficient in mitochondrial DNA (*ρ*0 cells), or cells treated with mito-TEMPO, fail to activate AMPK [4, 5] and abolishes the protective effects induced by ischemic conditioning further corroborates the concept that mitochondrial ROS is required to confer protection against ischemic insults [30].

## 4. The Diverse Effects of AMPK Activation on Ischemia, Ischemia-Reperfusion, and Preconditioning in the Brain

Some studies have shown that AMPK activation via ischemic preconditioning can prevent injury during ischemia-reperfusion in many organs including the heart, liver, and kidney [49, 55–58]. In contrast, investigators found that acute activation of AMPK prior to lethal ischemia is detrimental to the brain, whereas mild activation of AMPK signaling is beneficial [58–60]. Indeed, the authors of those studies demonstrated detrimental outcomes *in vivo* and *in vitro* as evident by the induction of larger infarct volumes, lower neurobehavioral scores, and decreased cell viability compared to control groups.

It is conceivable that the detrimental effects of acute and prolonged activation of AMPK prior to ischemia depend on both the metabolic status and mitochondrial health of neurons prior to ischemia. Given that neurons consume the majority of glucose (~20% of total glucose) and rely on oxidative phosphorylation to thrive, neurons predominantly utilize glucose as their main substrate for producing energy via oxidative metabolism [61]. However, neurons lack or contain very low levels of the 6-phosphofructo-2-kinase (PFK2) which is required to synthesize fructose-2,6-bisphosphate (F2,6P2), a powerful allosteric activator of PFK1 [62, 63]. Neurons can oxidize fatty acids and utilize amino acids. Hence, when ischemia ensues, p-AMPK will not lead to an increase in glycolysis but increased fatty acid oxidation in neurons, leading to enhanced oxidative phosphorylation to restore the ATP levels. Enhanced AMPK signaling in the brain under anaerobic conditions or hypoglycemia leads to metabolic failure during ischemia with detrimental consequences. Hence, increased AMPK signaling does not consistently protect neurons from ischemia insult as in other cells such as myocyte, hepatocyte, renal cell, endotheliocyte, or even adjacent astrocytes [11, 56, 57, 64].

Over the past decades, other investigators have made considerable efforts to illustrate the role of AMPK in cerebral ischemia. In order to avoid the off-target effects by drugs that activate AMPK, genetic models that ablate the expression for either the *α*1or *α*2 catalytic subunit of AMPK have been examined to further elucidate the *in vivo* role of AMPK in cerebral ischemia [65, 66]. Since AMPK activation likely enhances metabolism and survival of astrocytes as in peripheral tissues, increased AMPK activity can provide a favorable bioenergetics environment for neurons via the lactate shuttle [11]. Hence, future studies should explore whether AMPK can be activated specifically in CNS neurons. Indeed, a recent paper shed light on the effects of AMPK activation in neurons *in vivo* [67]. In brief, one study showed that AMPK is not activated in neurons during lethal ischemia phase but only during the ischemic preconditioning phase induced by cortical spreading depression (CSD) prior to the onset of ischemia. In addition, CSD enhances ischemic tolerance to temporary focal ischemia and a significant increase in the levels of phosphorylated *α* subunit of AMPK occurs 12 h. following CSD [67, 68]. The increased level of phosphorylated *α* subunit of AMPK was restricted to neurons—neurons predominantly located within the superficial layers of the cerebral cortex—but was not observed in astroglial cells. This observation was further confirmed by Shen et al. 2017 [67].

## 5. AMPK Can Be a Double-Edged Sword during Ischemia

Based on the aforementioned studies, we can conclude that acute AMPK activation prior to ischemia is protective in peripheral tissues but leads to a detrimental outcome in the brain [49, 56, 57, 64, 69, 70]. As mentioned before, these stark contradictions may be explained by the inherent energy metabolism conditions of neurons prior to the onset of toxic insults (e.g., AMPK increases oxidative phosphorylation during anaerobic conditions if activated during ischemia). However, the extent of activation of the AMPK-MFF-mitochondrial fission axis during ischemia may also contribute to these disparate effects [29, 40, 41]. Hence, future studies in *in vivo* models of ischemia are warranted to understand the role of the AMPK-MFF-fission pathway on neuronal survival in the context of ischemia.

However, there is a consensus that brief periods of AMPK activation prior to ischemia can enhance neuronal survival whereas sustained activation of AMPK induces cell death [11, 60, 67]. In addition, diverse stimuli that can lead to transient AMPK activation, such as brief glutamate exposure [71] and mild mitochondrial-uncoupling stimuli [72, 73]; brief periods of oxygen glucose deprivation *in vitro* [74]; or brief and intermittent blood vessel occlusion cycle *in vivo* [60], and CSD [67] can enhance tolerance to ischemia in an AMPK-dependent manner. AMPK activation in this manner prevents neurons from degenerating during ischemia or ischemia/reperfusion by initiating autophagy [67, 74], inducing translocation of glucose transporter 3 (GLUT3) [71], and promoting higher mitochondrial membrane potential to maintain Ca^+2^ homeostasis [75] or directly decrease AMPK levels in lethal ischemia [60]. Therefore, AMPK is an enticing target for eliciting neuroprotection via ischemia preconditioning in the brain despite its proapoptotic role during lethal ischemia. This concept is further elaborated below in Section 8. However, this phenomenon is just another example of the importance of ischemic preconditioning and reinforces the concept that **“**what does not kill you will make you stronger”, quoted by Annalisa Carlucciby Nietzsche.

## 6. D-AKAP1 Interacts with AMPK to Regulate Survival: Implications for Ischemia/Diabetes

D-AKAP1 (AKAP140/149 and other splice variants AKAP121, sAKAP84) is a protein scaffold that targets PKA to the outer mitochondrial membrane (OMM) to phosphorylate the proapoptotic protein BAD and the profission protein Drp1 [76, 77] to induce mitochondrial fusion and stabilize mitochondrial networks, a phenomenon that is associated with enhanced neuroprotection against toxic insults [28, 77, 78].

Recent evidence suggests that D-AKAP1 and AMPK interact to regulate mitochondrial function and structure. Indeed, D-AKAP1 is a substrate of AMPK. By using targeted *in vitro* AMPK screens and phosphorylation prediction algorithms, one recent study elegantly showed that D-AKAP1 is a substrate of AMPK in skeletal muscle cells [79]. The physiological effects of AMPK-mediated phosphorylation of D-AKAP1 include an increase in oxidative phosphorylation and mitochondrial-mediated *β* oxidation of lipids in L6 myoblasts. Mechanistically, AMPK phosphorylates D-AKAP1 in S103 to maintain mitochondrial respiration and transmembrane potential [79]. However, it remains to be seen whether AMPK phosphorylates D-AKAP1 in other tissues. Moreover, it remains to be known whether AMPK-mediated phosphorylation of D-AKAP1 affects mitochondrial dynamics and quality control. In addition, it is conceivable that AMPK uncouples PKA from D-AKAP1 to promote mitochondrial fission and mitophagy. Indeed, there is rationale and evidence that other signaling pathways can uncouple PKA from D-AKAP1 to promote fission. For instance, PINK1, a ser/thr mitochondrial kinase mutated in recessive forms of Parkinson's disease, triggers the displacement of PKA from D-AKAP1 during toxic insults that damage mitochondria and thereby ensures that fission of damaged mitochondria promotes mitophagy [80]. Hence, during oxidative stress, it is conceivable that AMPK-mediated phosphorylation of D-AKAP1 may limit the accessibility of PKA to D-AKAP1, presumably to allow AMPK to phosphorylate MFF as a feed-forward mechanism to promote mitochondrial fission. In summary, we raise the possibility that AMPK and D-AKAP1/PKA participate in a “tug of war” to regulate mitochondrial fission/fusion. For instance, increased AMPK signaling may induce mitochondrial fission by opposing D-AKAP1/PKA. The D-AKAP1-PKA-Drp1signaling axis promotes mitochondrial fusion whereas the AMPK-mitochondrial fission/fusion signaling axis leads to mitochondrial fragmentation and subsequent activation of mitophagy during toxic stress in neurons, both pathways leading to increased cell survival and mitochondrial homeostasis. However, in the absence of D-AKAP1/PKA signaling during oxidative stress, AMPK can act as a beneficial, compensatory signaling pathway to exert cytoprotection as elaborated below.

There is clear evidence that AMPK can converge at the mitochondrion to enhance protective D-AKAP1/PKA signaling. As reiterated, PKA-mediated phosphorylation of Drp1 via D-AKAP1 promotes mitochondrial fusion and prevents the activation of apoptosis in cells [28]. However, in models of diabetes and chronic stress, PKA-mediated phosphorylation of Drp1 is decreased, leading to mitochondrial fission [40, 81]. Treating cells with AMPK activators such as metformin or AICAR restores mitochondrial interconnectivity by enhancing PKA-mediated phosphorylation of Drp1 [49, 53]. However, additional studies are warranted to unveil the mechanism by which AMPK signaling interplays with mitochondrial PKA in models of ischemia and diabetes.

Beyond the mitochondrion, AMPK and PKA have been shown to cross-talk and interact in a feed-forward manner. For instance, treating vascular smooth muscle cells with AICAR not only stimulates AMPK signaling but also enhances PKA signaling [82]. However, the mechanisms by which these two holoenzymes interact, and whether this interaction occurs in cardiac cells, remain to be elucidated in future studies.

D-AKAP1 is a cardioprotective protein scaffold of PKA. D-AKAP1 is rapidly targeted for proteolytic degradation during oxidative stress via the E3 ligase Siah2 during ischemia [83, 84]. Chronic depletion of endogenous D-AKAP1, as evident in D-AKAP1 knockout mice, show aberrant mitochondrial structure as assessed by electron microscopy, increased ROS production, and reduced mitochondrial function following myocardial infarction (MI). These alterations were associated with robust cardiac mitophagy and apoptosis. Interestingly, reductions in D-AKAP1 levels are correlated with increased infarct size following myocardial infarction in D-AKAP1^−/−^ mice subjected to ligation of the left coronary artery [84]. However, this study did not address whether elevated mitophagy observed in D-AKAP1 knockout mice subjected to MI is phenocopied by AMPK activation. Consistent with this study, another study showed that a rat model of cardiac hypertrophy depletes D-AKAP1 levels in heart tissue, molecular pathology that is associated with mitochondrial dysfunction [85]. Furthermore, cardiac hypertrophy led to a decrease in endogenous levels of D-AKAP1 via downregulation of nuclear-localized cAMP signaling pathways and significantly increased ROS production [85].

**Figure 2:**
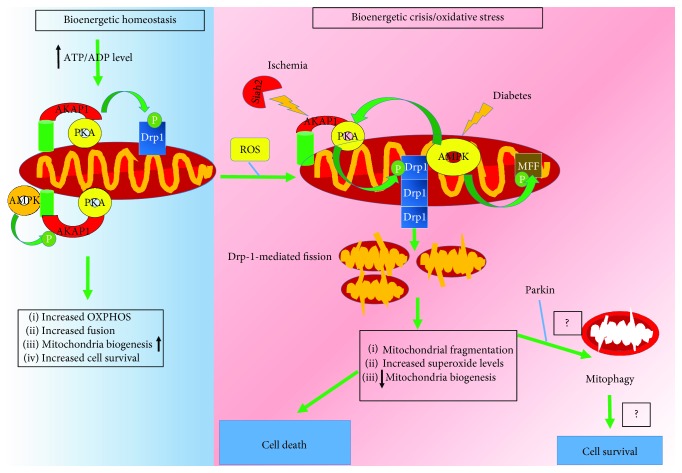
Model on how AMPK and D-AKAP1/PKA cooperate to regulate mitochondrial structure/function under physiological conditions and during oxidative stress induced by diabetes and ischemia. During homeostasis (indicated in shaded blue), D-AKAP1/PKA and AMPK regulate mitochondrial structure and function to maintain a high ATP/ADP ratio. D-AKAP1/PKA phosphorylates Drp1 at the OMM to inhibit its fission activity and, thereby, promote mitochondrial fusion and maintain oxidative phosphorylation. Concomitantly, AMPK phosphorylates D-AKAP1 leading to stable mitochondrial bioenergetics and structure through an unknown molecular mechanism. These posttranslational events lead to enhanced mitochondrial biogenesis and cell survival. On the other hand, acute or a transient increase in the level of oxidative stress (indicated in shaded red) leads to decreased kinase signaling (uncoupling of PKA from D-AKAP1) and decreased mitochondrial oxidative phosphorylation and mitochondrial dysfunction (decreased transmembrane potential), ensuing mitochondrial damage. AMPK phosphorylates MFF to promote mitochondrial fission, a cellular event that is associated with increased mitophagy. However, is not known whether increased AMPK-mediated fission enhances mitophagy or increased cell survival (conceptual gaps indicated by question marks). On the other hand, conditions that promote ischemia or chronic high levels of oxidative stress, as observed in models of diabetes and CVDs, leads to rapid degradation of endogenous D-AKAP1 through Siah2 (hypoxia), decreased AMPK signaling (diabetes models), increased superoxide levels, and decreased compensatory responses to replenish high quality mitochondria and eventual cell death.

Autophagy may be protective during ischemia, whereas it may be detrimental during reperfusion [86–88]. Thus, D-AKAP1 and AMPK stimulate mitochondria biogenesis and coregulate mitochondrial fission/fusion. Therefore, the interplay of these two proteins during oxidative stress may explain how these two ser/thr kinase govern ischemic preconditioning. Hence, we propose that maintaining normal levels of D-AKAP1 is necessary to preserve a pool of high quality and healthy mitochondria while low levels of D-AKAP1 may stimulate mitophagy, presumably via AMPK activation, of damaged mitochondria following ischemic conditioning (Figure 2).

## 7. The Dual Roles of AMPK Signaling in Diabetes

AMPK has dual effects on mitochondrial function and structure, which likely depends on the levels of oxidative stress of cells. Under physiological conditions, AMPK promotes mitochondrial fission, presumably to stimulate mitophagy as a protective mitochondrial quality control mechanism [29]. However, in chronically stressed cells, AMPK confers cytoprotection, presumably by promoting mitochondrial fusion, mitochondrial biogenesis, eliciting antioxidant responses, and restoring mitochondrial function [29, 89, 90]. Another study showed that enhancing AMPK signaling promotes mitochondrial fusion and reduces cell death caused by ischemia/reperfusion *in vivo* and *in vitro* [30].

Diabetes increases the risk for damaging endothelial cells and heart tissue. In addition, AMPK signaling is protective in several *in vivo* models of diabetes [91]. For instance, *in vivo* rodent models of diabetes exhibit decreased AMPK-mediated signaling (Thr-172 phosphorylation), in liver and kidney tissues [26]. Therefore, reduced AMPK signaling is associated with an inability of cells to mount necessary AMPK-mediated responses to compensate for the loss of energy and mitochondrial dysfunction (Figure 2). In cell cultures of diabetes, enhancing AMPK signaling can protect cells against hyperglycemia and hypoglycemia [91]. For instance, cell stress induced by hyperglycemia can elicit Drp1-mediated mitochondrial fission and increased mitochondrial superoxide, cytopathology that is reversed by elevating AMPK signaling [92]. In addition, pancreatic *β* cells maintained in low levels of glucose exhibited an increase in superoxide levels, decreased mitochondrial oxidative phosphorylation, and robust phosphorylation of AMPK and of AMPK substrates [31]. However, the physiological implications for AMPK activation in pancreatic *β* cells remain to be elucidated in this context.

Pharmacological activators of AMPK are cardioprotective in models of diabetes.

Indeed, the AMPK activator metformin efficiently reduces the steady-state levels of mitochondrial superoxide and mitochondrial fission in endothelial cells derived from streptozocin-treated rats, a well characterized *in vivo* model for diabetes [26]. The protective effects of metformin require AMPK activation as transfecting hyperglycemic HUVECs with a constitutively active mutant of AMPK phenocopies the ability of metformin for blocking Drp1-mediated mitochondrial fission and Drp1-mediated increased levels of mitochondrial superoxide [41]. Another study showed that rats treated with streptozocin for four weeks showed a marked reduction in the levels of superoxide, decreased mitochondrial biogenesis, decreased oxidative phosphorylation, and phosphorylation of AMPK in the heart and the kidney [26]. Pharmacologically cotreating streptozocin-treated mice with the AMPK activator AICAR restores AMPK signaling, reverses mitochondrial pathology, restores mitochondrial oxidative phosphorylation, and reverses kidney pathology, further supporting the concept that activating AMPK signaling is protective in models of diabetes [26] (Supplementary Materials online, Table S2). In another study, treating streptozocin/ApoE^−/−^ mice with metformin was able to reduce mitochondrial fragmentation [41].

Physiological activation of AMPK activation can protect the heart from ischemia by upregulating glucose uptake and energy-generating glycolytic pathways as well as enhancing fatty acid oxidation. Specifically, phosphorylation of phosphofructokinase [63] by AMPK can promote the generation of ATP *via* glycolysis [63]. However, downstream ischemia-protective pathways activated by AMPK are blunted in type 2 diabetes. In this context, heart tissue exhibits little flexibility to compensate for energy loss in a similar manner to what neurons undergo during ischemia (Figure 2). A high body mass index and obesity are risk factors for developing CVDs and diabetes. Interestingly, endogenous mRNA levels of D-AKAP1 and of type II regulatory subunit of PKA (PKA/RII*β*) have been observed to be decreased in adipocytes and subcutaneous adipose tissue of obese individuals [93, 94]. Although is not known whether D-AKAP1 levels are downregulated in tissues of diabetes individuals, it is worth noting that D-AKAP1 is transcriptionally regulated by PPR*γ* in a PKA-dependent manner [93]. Therefore, given that PPR*γ*-mediated signaling is impaired in type II diabetes, these observations suggest that decreased expression of D-AKAP1 and PKA/RII*β*—proteins involved in lipolysis and mitochondrial metabolism—may contribute to the pathology in CVDs and type II diabetes subjects with a high BMI. In addition, patients with type 2 diabetes (T2D) are highly susceptible to developing CVD and restoring normoglycemia alone is insufficient for reducing the risk of CVDs in T2DM [95] suggesting that other therapeutic strategies need to be developed to reduce the onset of cardiovascular complications. In support of this concept, several known antidiabetic drugs in clinical work are used to reduce the incidence of diabetes-related CVDs such as metformin, thiazolidinediones (TZDs), and statins. Indeed, Cilostazol has been shown to restore AMPK activation and exert cardio- and vasculoprotective actions in *in vivo* or *in vitro* [96–102].

## 8. Therapeutic Perspectives

Diabetic individuals are highly prone to experiencing strokes, minitransient ischemic episodes, and other cerebral vascular complications [91]. As mentioned before, there is a consensus that AMPK is protective in models of diabetes. Given that the levels of AMPK signaling are severely compromised in diabetic tissues [26, 103], normalizing AMPK activity—as induced by metformin—continues to be an enticing therapeutic strategy for treating diabetes. On the other hand, it is not clear whether eliciting AMPK signaling is protective in models of ischemia. Some studies have shown that AMPK is protective during the ischemic phase in mice subjected to ischemia/reperfusion paradigms [104]. *In vitro*, pharmacologically pretreating tissues with AMPK activators can protect in cell culture and *in vivo* models of ischemia [15, 105, 106] (Supplementary Materials online, Table S2). Conversely, one *in vivo* study showed that inhibitors of AMPK activity increased protection against brain damage following ischemia [107]. Hence, future studies are warranted to further identify specific “windows” of opportunity by which AMPK confers robust neuroprotection during ischemia.

### 8.1. D-AKAP1: An AMPK Substrate with Therapeutic Applications

Overall, the aforementioned published data suggests that PKA and AMPK converge in the mitochondrion to enhance cytoprotection against ischemia. However, as mentioned before, how mitochondrial PKA (D-AKAP1/PKA) and AMPK cooperate to regulate survival of neurons or cardiac cells depends on the oxidative status of cells. During chronic stress, which may decrease PKA signaling in mitochondria, AMPK may serve to promote mitophagy. However, during physiological conditions, both kinases may cooperate to maintain mitochondrial function and survival. Consistent with the concept that AMPK and mitochondrial PKA cooperate to maintain cell survival during ischemia, one study demonstrated that PKA-mediated phosphorylation of Drp1 and PKA-mediated mitochondrial fusion during nitrite-preconditioning conditions exert cytoprotection of cardiac myocytes against ischemia *in vivo* and *in vitro* [30]. Nitrite-induced PKA-mediated protection of cardiac cells against ischemia requires AMPK activity and mitochondrial ROS [30]. Hence, given that AMPK signaling is blunted in diabetes, these studies suggest that therapeutic interventions that activate mitochondrial PKA can be beneficial to prevent the onset of ischemia in diabetes.

D-AKAP1 is highly sensitive to proteolytic degradation via the E3 ubiquitin ligase Siah2 during ischemia [83, 108]. Given that D-AKAP1 robustly and rapidly undergoes Siah2-mediated degradation during ischemia, it is conceivable that small molecular compounds that can coactivate PKA or stabilize endogenous levels of D-AKAP1 may confer significant protection during ischemia. There is experimental evidence to support this notion. For instance, Siah2 knockout mice exhibit little heart pathology after being subjected to ischemia reperfusion (e.g., myocardial infarct size) [108]. Cells treated with diffusible cyclic AMP analogues can increase the expression of D-AKAP1 in cell culture studies [109]. Therefore, it is conceivable that other PKA activators (e.g., forskolin) could be employed to increase D-AKAP1 *in vivo* to prevent the degradation of D-AKAP1 during ischemia.

There is significant controversy on the consequences of global activation of PKA on cell survival during ischemia. For instance, overt PKA activation exacerbates pathology in heart tissue during ischemia. Indeed, excessive *β*-adrenergic receptor activation leads to activation of protein kinase A (PKA), leading to increased opening of the L-type calcium currents and a subsequent increase in cytosolic calcium levels, the latter being potentially harmful to cardiac tissue [110, 111]. On the other hand, treating ischemic heart tissue with H89, an inhibitor of protein kinase A, promotes postischemic cardiac contractile recovery and reduces infarct size [112]. In neurons, the binding of cyclic AMP to the regulatory subunit of PKA is rapidly inhibited during the acute phase of cerebral ischemia, leading to reduced neuronal survival [113–115]. Hence, in this case, enhancing PKA signaling prior to ischemia may prove beneficial.

In addition, activation of global PKA activity can negate the protective effects of AMPK in different models of chronic stress and degeneration. For instance, while different studies have shown that PKA governs AMPK-mediated mitochondrial biogenesis and cytoprotection [25, 116], cytosolic PKA has been shown to oppose various physiological effects of AMP kinase in insulin-signaling cells and blocks the ability of metformin for decreasing glucose levels in primary hepatocytes [117, 118].

Given that D-AKAP1 is a substrate of AMPK, it is conceivable that compounds that elicit AMPK-mediated phosphorylation of D-AKAP1 are a better therapeutic option as opposed to using global PKA activators (e.g., forskolin) for conferring neuroprotection during ischemia. On the other hand, AMPK-activating compounds such as AICAR increases PKA signaling *in vitro* [82]. Hence, it remains to be seen whether cotreatment of heart cells or neurons with AMPK and PKA-stimulating compounds exert an additive, protective effect during ischemia. Alternatively, compounds that increase the endogenous levels of D-AKAP1 can offer cytoprotection in models of ischemia. Experimentally induced cardiac infarct depletes D-AKAP1 levels, molecular pathology that is associated with mitochondrial dysfunction [85]. In that study, cyclic AMP analogues were used to increase endogenous D-AKAP1 levels to protect cardiac cells from ischemia insult. To this end, it is conceivable that pharmacological compounds that increase D-AKAP1 levels can be used in hypertensive individuals with left ventricular hypertrophy. Therefore, we raise the possibility that D-AKAP1/PKA and AMPK are novel therapeutic targets for treating ischemia. However, future studies are warranted to elucidate whether single or dual pharmacological activation of mitochondrial PKA or AMPK can confer protection during ischemia.

## Supplementary Materials

Supplementary materials Table S1: Different conditions that induce AMPK activation through ROS. Table S2: Other conditions that activate AMPK in a non-ROS-mediated mechanism.
