# Supplementary material for: How AMPK and PKA Interplay to Regulate Mitochondrial Function and Survival in Models of Ischemia and Diabetes
Source: Oxid Med Cell Longev. 2017 Dec 17;2017:4353510. doi: 10.1155/2017/4353510 (PMC5748092; doi:10.1155/2017/4353510)
Supplement: Supplementary materials — Table S1: Different conditions that induce AMPK activation through ROS. Table S2: Other conditions that activate AMPK in a non-ROS-mediated mechanism. [file 4353510.f1.docx]

**SUPPLEMENTARY MATERIAL**

**AMPK and PKA regulate mitochondrial function and survival against oxidative stress in models of ischemia and diabetes**

Jingdian Zhang^a^, Yumeng Wang^b^, Xiaofeng Liu^c^, Ruben K. Dagda^d^＆Ying Zhang^a^,

^a^Department of Neurology and Neuroscience Center, First Hospital of Jilin University, Xinmin

Street No 71, Changchun 130000, China ; ^b^Department of Physiology, College of Basic Medical Sciences, Norman Bethune Health Science Center , Jilin University Xinmin Street No 126, Changchun 130000, China; ^c^Neuroscience Research Center, the First Hospital of Jilin University, East democracy Street No 519, Changchun 130000, China; ^d^ Department of Pharmacology, University of Nevada, Reno School of Medicine, Mailstop 318, Manville Health Sciences Building 19A(Office)/18, Reno, NV, 89557

**Table S1**: **Different conditions that induce AMPK activation through ROS**

| Situations/drugs | Cell/tissue type | Treatment details | Source  of  ROS | Measures to  confirm ROS  source | | Upstreamsignals of AMPK activation | | Subunit of AMPK | | References |  |  |
| --- | --- | --- | --- | --- | --- | --- | --- | --- | --- | --- | --- | --- |
| Hypoxia | AMPKαWT and AMPKα1-/-2-/- mouse embryonic fibroblasts | hypoxic conditions (1.5% O2) | mtROS:complex III | | Antioxidant EUK-134 and  ρ^0^ 143B cells  and  ΔCytochrome b cell | | LKB1 | | α | ([Emerling *et al.*, 2009](#_ENREF_1); *Free radical biology & medicine*, **46**, 1386-1391) | |  |
| Berberine  Nitrite | HUVECs and BAEC  H9c2 cardiomyocyte Isolated-perfused heart | 10 mM of Berberine incubate for 2h  Transient(30min) normoxic nitrite treatment | mtROS: O2 ^–^and ONOO^-^  Mitochondrial superoxide | | β^0^-BAEC SOD1overexpression  ONOO^-^scavenger Mito-TEMPO  MitoTEMPO catalase overexpresβ^0^- H9c2 | | LKB1  * | | α  α1 | ([Han *et al.*, 2010](#_ENREF_2); PLOS One; 5: e15420)  ([Kamga Pride *et al.*, 2014](#_ENREF_3); *Cardiovascular research*, **101**, 57-68) | |  |
| Sevoflurane | Isolated Langendorff-perfused rat hearts | 3 times 5-minute episodes of sevoflurane(2.5vol%)prior I/R | ONOO^-^ | | OS-scavenger n-(2-mercaptopropionyl)-glycine | | * | | α | ([Lamberts *et al.*, 2009](#_ENREF_4); Circulation; **120**:S10-15) | |  |
| Starvation | HeLa ρ° and wt cells | Starvation of glucose, L-glutamine, pyruvate, serum | Mito-regulated ROS and specifically O2^-^ | | Overexpression SOD2  HeLa ρ° cells | | Not LKB1 in Hela | | α | ([Li *et al.*, 2013](#_ENREF_5); *Cell Signal*, **25**, 50-65.) | |  |
| Low glucose | INS1E cells | incubation at 1mMglucose | Mito-complex I | | Rotenone , myxothiazol ,MnTBAP, Mito-Tempol, | | Unknown kinase | | **α** | ([Sarre *et al.*, 2012](#_ENREF_6); Free Radical Biology and Medicine;52:142-150 ) | |  |
| Thyroid hormone T3 | Male SD rat | a single ip dose of 0.1 mgT3/kg | ROS | | antioxidant NAC | | CaMKKβ TAK1 | | α | ([Videla *et al.*, 2014](#_ENREF_7); *World journal of gastroenterology*, **20**, 17416-17425) | |  |
|  |  |  |  | |  | |  | |  |  | |  |

**Table S2** : **Other conditions that activate AMPK in a non-ROS-mediated mechanism**

| Measures/Activators | Mechanism of AMPK activation | Tissue/organ type | Ischemia type /outcome | Downstream effects of AMPK activation | reference |  |
| --- | --- | --- | --- | --- | --- | --- |
| **Pharmacological**  Metformin | AMP/AATP ratio | Male S-D rat brain cortex | pMCAO/ reduce infarct volume, neurological deficits and cell apoptosis | autophagy | ([Jiang *et al.*, 2014](#_ENREF_3); *British journal of pharmacology*, **171**, 3146-3157.) |  |
|  |  |  |  |  |  |  |
| AICAR | AMP/ATP ratio | Male Wistar rats heart | Regional ischemia of isolated perfused heart for 45 min/increases myocardial glucose uptake, reduced infarct size | glucose uptake | ([Kristiansen *et al.*, 2009](#_ENREF_5); *Basic & clinical pharmacology & toxicology*, **105**, 10-16) |  |
| A-769662 | binding to the β subunit | C57Bl6 AMPK KD mice /WT littermates mice heart | Isolated mouse heart perfusion and *in vivo* coronary occlusion/reperfusion/better ventricular contractile function | Inhibit eEF2 while activate ACC | ([Kim *et al.*, 2011](#_ENREF_4); *Journal of molecular and cellular cardiology*, **51**, 24-32). |  |
| **Physiological** |  |  |  |  |  |  |
| Exercise | AMP/ATP ratio | Male S-D rat brain cortex | tMCAO/ reduce infarct volume,neurological deficits and neuron apoptosis | PFK | ([Dornbos *et al.*, 2013](#_ENREF_1); *Journal of neuroscience research*, **91**, 818-827) |  |
| Calorie restriction | AMP/ATP ratio | Fischer-344rats heart | Langendorff-perfusion of hearts and Myocardial ischemia/reperfusion/ improved the recovery of LV function | * | ([Shinmura *et al.*, 2005](#_ENREF_9); *Journal of molecular and cellular cardiology*, **39**, 285-296. ) |  |
| **Natural compounds** |  |  |  |  |  |  |
| Resveratrol | * | Male S-D rat brain cortex | tMCAO 2h/ reduce infarct volume, neurological deficits and promotes restoration of brain tissue ATP levels | cAMP/AMPK/SIRT1 | ([Wan *et al.*, 2016](#_ENREF_11); *Brain research bulletin*, **121**, 255-262) |  |
| Salvianolic Acid | AMP/ATP ratio | Male S-D rat brain cortex | tMCAO 1h/attenuated I/R-induced microcirculatory, neuronal damage | Inactivate NADPH oxidase by AMPK/Akt/PKC | ([Tang *et al.*, 2014](#_ENREF_10); *Microcirculation*, **21**, 615-627) |  |
| **Other measures** |  |  |  |  |  |  |
| Cortical spreading depression | * | Male Wistar rat brain | tMCAO 2h/ significantly decreased the infarct volume, neurological deficits and neuronal apoptosis | autophagy | ([Shen *et al.*, 2016](#_ENREF_8); *Journal of neurochemistry*.140:(5), 799-813) |  |
| Remote Ischemic  Conditioning | * | C57BL/6 mice heart | global ischemia using a Langendorff preparation/ reduce Infarct size, improvements in post-ischemic left ventricular end diastolic pressure (LVEDP) and developed pressure (LVDP) | autophagy | (Rohailla *et al.*, 2014; *PloS one*, **9**, e111291) |  |
| Intraventricular balloon and aorto-caval shunt (ACS)stretch | * | Male S-D rat heart | global ischemia using Langendorff preparation/ reduce myocardial infarct, improved post-ischemic recovery | ACC | (Hao *et al.*, 2010; *The Korean journal of physiology & pharmacology*  **14**, 1-9.) |  |
| Electro-acupuncture | * | male C57BL/6 mice hippocampus | BCCAO 15 minutes/ reduce cell apoptosis, less hippocampal neuronal morphology disorders | * | (Ran *et al.*, 2015 *Neural regeneration research*, **10**, 1069-1075) |  |
|  |  |  |  |  |  |  |
|  |  |  |  |  |  |  |
